# Diverse Physiological Functions of FAB1 and Phosphatidylinositol 3,5-Bisphosphate in Plants

**DOI:** 10.3389/fpls.2019.00274

**Published:** 2019-03-22

**Authors:** Tomoko Hirano, Masa H. Sato

**Affiliations:** Graduate School of Life and Environmental Sciences, Kyoto Prefectural University, Kyoto, Japan

**Keywords:** Arabidopsis, phosphoinositides, biological membranes, PtdIns(3,5)P_2_, PtdIns-binding proteins

## Abstract

Biological membranes are predominantly composed of structural glycerophospholipids such as phosphatidylcholine, phosphatidylethanolamine, and phosphatidylinositol. Of the membrane glycerophospholipids, phosphatidylinositol (PtdIns) and its phosphorylated derivatives (phosphoinositides) constitute a minor fraction yet exert a wide variety of regulatory functions in eukaryotic cells. Phosphoinositides include PtdIns, three PtdIns monophosphates, three PtdIns bisphosphates, and one PtdIns triphosphate, in which the hydroxy groups of the inositol head group of PtdIns are phosphorylated by specific lipid kinases. Of all the phosphoinositides in eukaryotic cells, phosphatidylinositol 3,5-bisphosphate [PtdIns(3,5)P_2_] constitutes the smallest fraction, yet it is a crucial lipid in animal and yeast membrane trafficking systems. Here, we review the recent findings on the physiological functions of PtdIns(3,5)P_2_ and its enzyme—formation of aploid and binucleate cells (FAB1)—along with the regulatory proteins of FAB1 and the downstream effector proteins of PtdIns(3,5)P_2_ in *Arabidopsis*.

## Introduction

Biological membranes are physical barriers that regulate cellular biological reactions through dedicated permeable zones. They are composed of major structural glycerophospholipids such as phosphatidylcholine and phosphatidylethanolamine alongside minor regulatory phospholipids such as phosphatidylinositol (PtdIns) and its phosphorylated derivatives (phosphoinositides), which exert a wide variety of regulatory functions in all eukaryotic cells. Phosphoinositides spatiotemporally regulate diverse downstream cellular pathways *via* the recruitment of various effector proteins through specific membrane domains (Balla, 2013). Phosphatidylinositol 3,5-bisphosphate [PtdIns(3,5)P_2_] is the least abundant phosphoinositide in eukaryotic cells, comprising approximately 0.05–0.1% of the total phospholipids. Studies have shown that various mammalian physiological signals such as hormones and growth factors, and osmotic or oxidative stress signals in yeast and plant cells, cause a rapid elevation in PtdIns(3,5)P_2_ levels (Dove et al., 1997; Meijer et al., 1999; Sbrissa et al., 1999; Bridges et al., 2012; Hirano et al., 2015). PtdIns(3,5)P_2_ levels are regulated by a synchronized mechanism consisting of PtdIns 3-phosphate 5-kinase, formation of aploid and binucleate cells/FYVE finger-containing phosphoinositide kinase (FAB1/PIKfyve), and a PtdIns(3,5)P_2_-phosphatase (SAC3/FIG 4) (Hasegawa et al., 2017). FAB1 and PIKfyve are localized in the vacuoles and endolysosomes, respectively, and carry out essential roles in endosomal membrane trafficking, including vacuolar sorting, endocytosis of membrane proteins, ion transport, cytoskeleton dynamics, and retrograde transport in animals and yeasts (Efe et al., 2005; Shisheva, 2008; Ikonomov et al., 2011). PtdIns(3,5)P_2_ is the product of FAB1, and has crucial roles in maintaining membrane trafficking, autophagy, and signaling mediation in response to various cellular stresses (Shisheva, 2008). It has been demonstrated that loss of FAB1/PIKfyve function causes severe defects during embryogenesis, resulting in embryonic lethality in *Drosophila* spp., *Caenorhabditis elegans*, and *Mus musculus* (Nicot et al., 2006; Rusten et al., 2006; Ikonomov et al., 2011; Takasuga et al., 2012).

The majority of eukaryotes contain a single copy of the FAB1-encoding gene; however, *Arabidopsis* has four distinct FAB1-related genes (FAB1A, FAB1B, FAB1C, and FAB1D) of which only FAB1A and FAB1B contain a conserved FYVE (FAB1, YOTB, Vac1, and EEA1) domain (Mueller-Roeber and Pical, 2002). The diversity of FAB1 genes indicates the vast array of functions that FAB1 and PtdIns(3,5)P_2_ have in *Arabidopsis* and higher plants. This review summarizes the current findings on the physiological roles of PtdIns(3,5)P_2_, its catalyst enzyme FAB1, and its regulating and effector proteins in *Arabidopsis*.

## Structures of FAB1/PIKfyve Complexes in Yeast, Mammals, and *Arabidopsis*

FAB1/PIKfyve forms a large protein complex with several regulatory proteins to simultaneously catalyze PtdIns 3-phosphate kinase and PtdIns(3,5)P_2_ phosphatase reactions, thereby regulating phosphoinositide 3,5-bisphosphate levels.

In yeast, the FAB1/PIKfyve complex is localized to the vacuole membrane and is composed of Fab1p, Fig 4p (Gary et al., 2002), an adaptor-like protein (Vac14p) (Bonangelino et al., 2002), and a FAB1 regulatory protein (Vac7p) (Bonangelino et al., 1997). FIG 4 is a phosphoinositide phosphatase enzyme that specifically catalyzes the production of PtdIns3P from PtdIns(3,5)P_2_ (Gary et al., 2002; Rudge et al., 2004; Duex et al., 2006a,b). The loss of FIG 4 function impairs the phosphatase and PtdIns3P 5-kinase activity of the FAB1 complex (Rudge et al., 2004; Duex et al., 2006b; Botelho et al., 2008), suggesting that FIG 4 may have an additional regulatory role in PtdIns(3,5)P_2_ synthesis. The adaptor-like protein Vac14p consists of multiple HEAT repeat arrays (Andrade and Bork, 1995) over almost its entire sequence (Jin et al., 2008), and forms oligomers *via* its C-terminal region (Dove et al., 2002; Jin et al., 2008; Alghamdi et al., 2013). Vac14p associates with all of the FAB1 complex proteins, acting as a scaffold protein, and it has been proven that the association of Vac14p with Fig 4p is necessary for the positive regulation of PtdIns(3,5)P_2_ synthesis (Duex et al., 2006a,b; Botelho et al., 2008; Jin et al., 2008). The FAB1 regulatory protein Vac7p is the only transmembrane protein with no conserved motif and no known metazoan homologs (Gary et al., 2002). Vac7p and Vac14p have been demonstrated to independently regulate PtdIns(3,5)P_2_ levels in yeast (Duex et al., 2006b).

In metazoan cells, FAB1 (also called PIKfyve in mammals) (Sbrissa et al., 1999) is localized in the early and late endosomes where it forms a complex with VAC14 (ArPIKfyve in mammals) (Sbrissa et al., 2004) and SAC3 (FIG 4) (Sbrissa et al., 2007; Ikonomov et al., 2013). The triple complex composed of PIKfyve (FAB1), ArPIKfyve (Vac14), and SAC3 (FIG 4) is known as the PAS complex, and regulates the synthesis and turnover of PtdIns(3,5)P_2_ (Sbrissa et al., 2007, 2008; Ikonomov et al., 2009).

**Figure 1:**
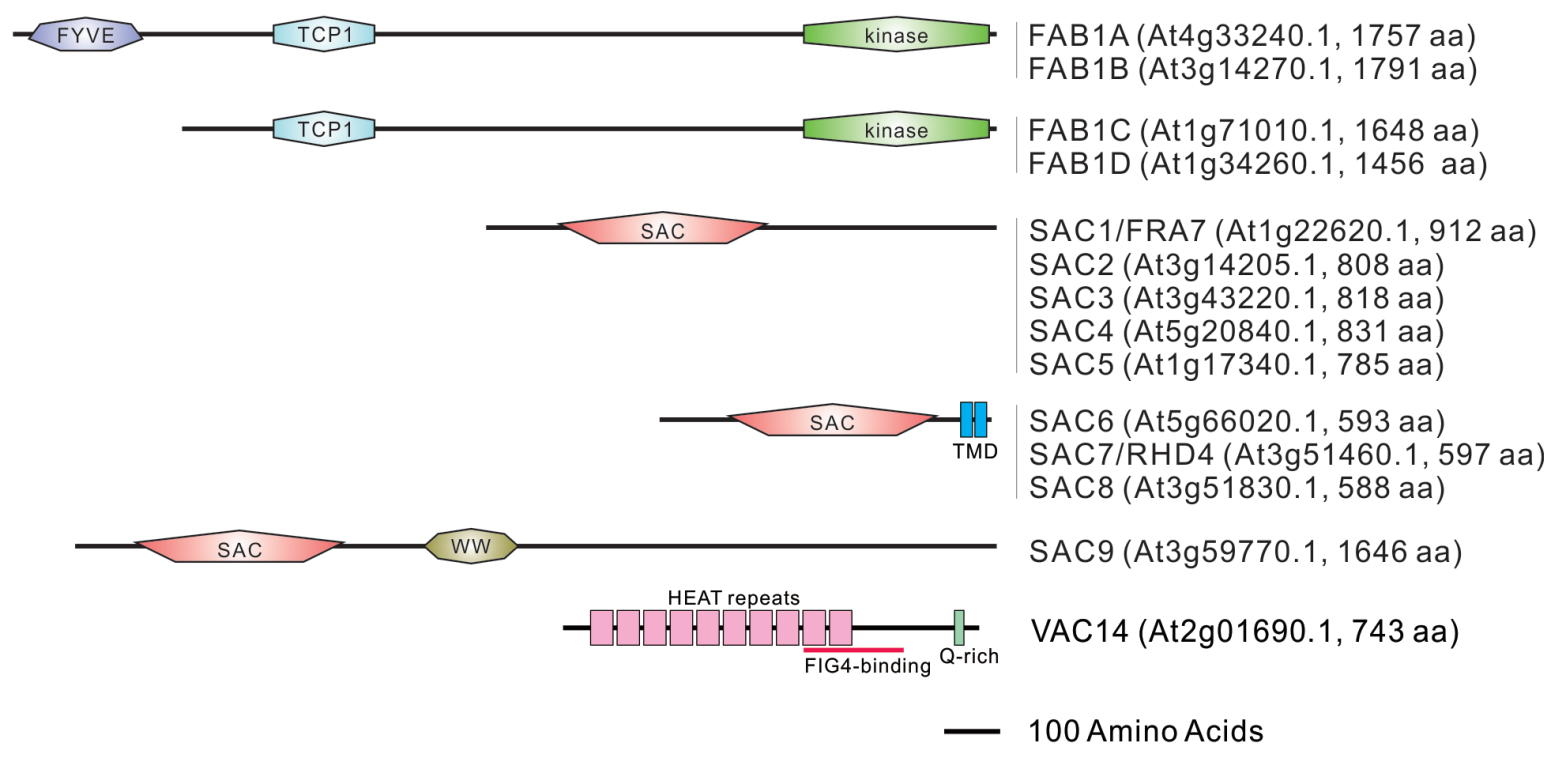
Domain structures of FAB1, SAC family proteins, and VAC14 in *Arabidopsis*. Domain structures were identified using the PROSITE program (https://prosite.expasy.org/). FYVE; FAB1, YOTB, VAC1, EEA1 zinc finger domain, TCP1; Tailless Complex Polypeptide 1 domain found in chaperone proteins, SAC; suppressor of actin domain, TMD; transmembrane domain, WW; a domain containing two conserved tryptophans, HEAT repeats; a tandem repeat structural motif composed of two alpha helices linked by a short loop found in Huntingtin, Elongation factor 3, protein phosphatase 2A, TOR1, Q-rich; a glutamine rich domain.

The *Arabidopsis* genome encodes four types of FAB1 (FAB1A–FAB1D), named based on their similarity to the yeast FAB1 (Whitley et al., 2009). Yeast FAB1 and mammalian PIKfyve have a conserved N-terminal FYVE (FAB1p, YOTB, Vac1p, and EEA1)-finger domain, the central part of which comprises a Cpn_TCP1 (HSP chaperonin T-complex protein 1) homology domain and the C-terminal of which comprises a kinase catalytic domain. The FYVE-finger domain is responsible for binding PtdIns3P and localizing it to the endosomes (Shisheva, 2008). *Arabidopsis* FAB1A and FAB1B also possess a conserved FYVE domain, and the *fab1afab1b* double mutant has a male gametophyte-lethal phenotype suggesting functional redundancy between FAB1A and FAB1B in *Arabidopsis*. Phylogenic analysis has indicated that FAB1 orthologs without the FYVE domain are clustered in the higher plant lineage (Whitley et al., 2009). Since the expression of FAB1C and FAB1D has been detected in various tissues (Bak et al., 2013; Serrazina et al., 2014), these proteins are likely to have diversified during the evolution of higher plants where they may have acquired novel functions beyond the canonical FAB1 (FAB1A and FAB1B) proteins. FAB1B and FAB1D have complementary roles in the regulation of membrane recycling, vacuolar pH, and the homeostatic ROS control in pollen tube growth, despite the cytosolic localization of FAB1D in the pollen tube (Serrazina et al., 2014). Unlike the gene arrangement of FAB1, *Arabidopsis* has a single VAC14 gene (Zhang et al., 2018). Bimolecular fluorescence complementation has revealed that FAB1A/B and VAC14 physically interact to form a functional protein complex in *Arabidopsis* (Whitley et al., 2009; Hirano et al., 2011; Zhang et al., 2018). The *Arabidopsis* genome includes 9 SAC-domain containing phosphatase (SAC1-SAC9) genes (Zhong and Ye, 2003) which can be separated into three different classes based on their homology; SAC1-SAC5 are the most similar to yeast FIG4p, SAC6-SAC8 have two C-terminal transmembrane domains and are the most similar to yeast SAC1p, and SAC9 is the largest protein containing a unique WW domain (Zhong and Ye, 2003) (Figure 1).

## Subcellular Localization FAB1 and PtdIns(3,5)P_2_ in *Arabidopsis*

In yeast and mammalian cells, the FAB1/PAS complex (comprising of FAB1, VAC14, & FIG 4) is localized in the vacuoles, endosomes, and lysosomes (Gary et al., 1998; Shisheva et al., 2001; Rudge et al., 2004; Botelho et al., 2008; Jin et al., 2008; Zhang et al., 2012). In *Arabidopsis*, FAB1A is predominantly localized in the SORTING NEXIN-1 (SNX-1)-residing late endosomes of the developmental cell division, transition, and elongation zones of epidermal and cortical cells (Hirano et al., 2015), whereas FAB1B localizes to the endomembrane compartments including endoplasmic reticulum (ER)-like reticulate structures and vacuoles of the pollen tubes. However FAB1D, the plant-specific FAB1 ortholog with no FYVE-domain, is mainly localized in the cytosol indicating that the N-terminal FYVE-domain of FAB1 is necessary for its endosomal localization in *Arabidopsis* (Serrazina et al., 2014).

The fluorescent PtdIns(3,5)P_2_-specific probe, based on tandem repeats of the cytosolic PtdIns(3,5)P_2_-interacting domain (ML1N) of the mammalian lysosomal transient receptor potential cation channel, Mucolipin 1 (TRPML1), was developed to label mammalian (Li et al., 2013) and *Arabidopsis* cells. Using this probe, PtdIns(3,5)P_2_ was predominantly observed on late endosomes in root cells. Unlike the yeast and mammalian cells, the vacuolar localization of the PI(3,5)P_2_-specific probe was never observed in *Arabidopsis* (Hirano et al., 2017b).

**Figure 2:**
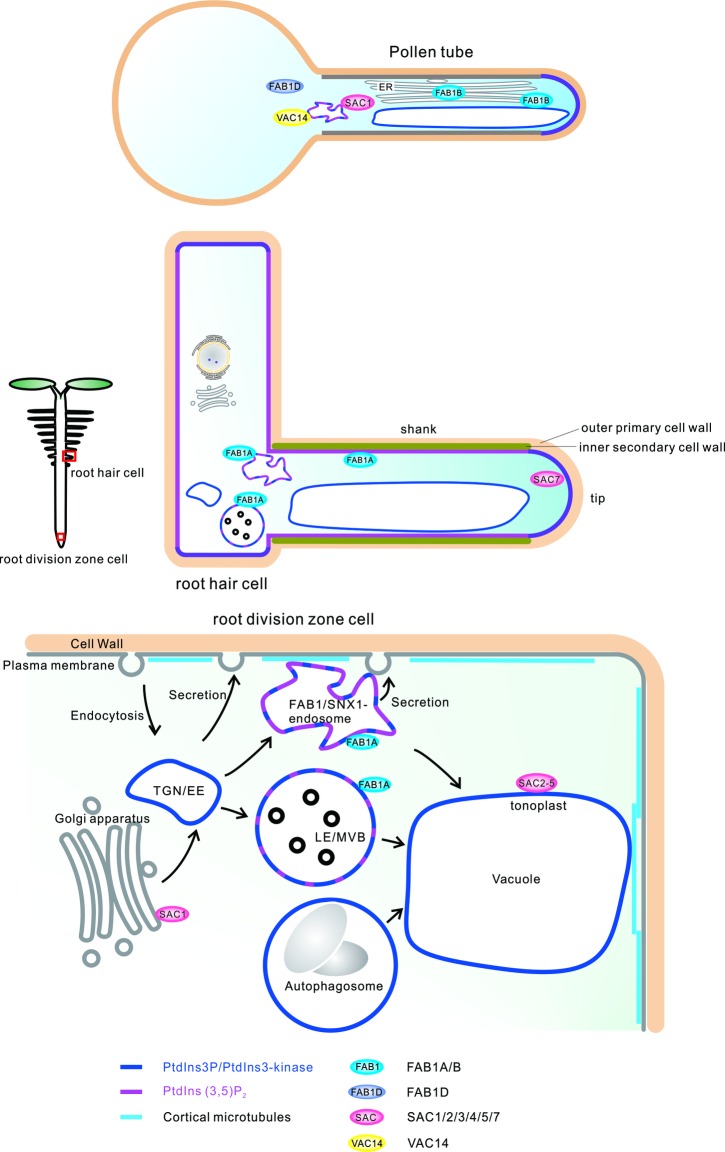
Subcellular localization of PtdIns3P, PtdIns(3,5)P_2_, FAB1s, SACs, and VAC14 in developing and mature *Arabidopsis* cells.

Intriguingly, FAB1A and PtdIns(3,5)P_2_ have been shown to be localized in the plasma membrane on the shank of growing root hairs, hardening the region by constructing a secondary cell wall and cortical microtubule structures. This suggests that FAB1 and PtdIns(3,5)P_2_ have acquired a novel function whereby they harden the root hair cell wall to regulate the organization of cortical microtubules and secretion of secondary cell wall structural components in higher plants (Hirano et al., 2018) (Figure 2).

## Physiological Functions of FAB1 and PtsIns(3,5)P_2_ in *Arabidopsis*

The most striking feature of PtdIns(3,5)P_2_ deficiency or FAB1(PIKfyve) dysfunction in many eukaryotic cells is the enlargement of vacuoles, endosomes, or lysosomes (Yamamoto et al., 1995; Ikonomov et al., 2001; Rutherford et al., 2006; Jefferies et al., 2008; Whitley et al., 2009; Hirano et al., 2011; Takasuga et al., 2012). In mammalian cells, impaired FAB1(PIKfyve) function has been reported to cause severe defects during embryogenesis, resulting in embryonic lethality in animals (Nicot et al., 2006; Rusten et al., 2006; Ikonomov et al., 2011; Takasuga et al., 2012). Furthermore, the *fab1afab1b* revealed a lethal male gametophyte phenotype in *Arabidopsis* (Whitley et al., 2009), with mutant pollen showing severe vacuolar reorganization and vacuolar acidification defects following the first mitotic division (Whitley et al., 2009). The inhibition of PtdIns(3,5)P_2_ production reduced vacuolar acidification and convolution, and delayed stomatal closure in response to ABA. Since vacuolar H^+^-pyrophosphatase has been shown to bind PtdIns(3,5)P_2_ *in vitro*, the authors hypothesized that PtdIns(3,5)P_2_ may stimulate the H^+^-pumping activity of vacuolar H^+^-pyrophosphatase in ABA-dependent stomatal closure (Bak et al., 2013). However, a patch-clamp study of the *Arabidopsis* vacuole demonstrated that nanomolar levels of PI(3,5)P_2_ regulate chloride channel a (CLC-a), a member of the anion/H^+^ exchanger family, which is implicated in stomatal movements in *Arabidopsis*, but not H^+^-pyrophosphatase (Carpaneto et al., 2017). These observations suggest that PtdIns(3,5)P_2_ is localized in the vacuolar membrane where it exerts various vacuolar functions to regulate PtdIns(3,5)P_2_ effector proteins, however the presence of PtdIns(3,5)P_2_ on the vacuolar membrane could not be detected by ML1N-based fluorescent probably due to the detection limit of the probe (Kd of GFP-ML1N*2 was calculated to be 5.6 μM (Li et al., 2013)).

The conditional down-regulation of *FAB1A* and *FAB1B* expression has been shown to cause various abnormal phenotypes including growth inhibition, hypo-sensitivity to exogenous auxin, disturbance of root gravitropism, and floral organ abnormalities. These pleiotropic developmental phenotypes are likely related to auxin signaling in *Arabidopsis* (Hirano et al., 2011). Auxin signaling defects in *fab1afab1b* knockdown are caused by the abnormal localization of the PIN2 and AUX1 auxin transporters, likely due to the inhibition of the late endosomal maturation process (Hirano and Sato, 2011; Hirano et al., 2015, 2017a). It has also been reported that conditional knockdown of *FAB1A* and *FAB1B*, or inhibition of PtdIns(3,5)P_2_ production using a FAB1 inhibitor (YM201636), induces the release of late endosomes from cortical microtubules and disturbs cortical microtubule organization, highlighting the importance of late endosome association in proper cortical microtubule construction (Hirano et al., 2015). Studies have shown although FAB1C lacks the conserved N-terminal FYVE domain required to bind PtdIns3P, it still successfully produces PtdIns(3,5)P_2_ from PtdIns3P *in vitro* (Bak et al., 2013). In addition, *fab1b* and *fab1c* T-DNA mutants exhibit stomatal closure defects, implying that FAB1B and FAB1C have overlapping functions in stomatal closure to control PtdIns(3,5)P_2_ levels in *Arabidopsis* guard cells (Bak et al., 2013). In *fab1b* and *fab1d* single mutants, pollen viability, germination, and tube morphology were not significantly affected, although the pollen tubes of these mutants were found to exhibit altered membrane recycling, vacuolar acidification, and decreased reactive oxygen species (ROS) production (Serrazina et al., 2014). Lack of the N-terminal FYVE-domain in FAB1C and FAB1D may confer different subcellular localization patterns of these proteins in plant cells. In fact, a subcellular localization prediction program (SUBA4) predicts the nuclear localization of FAB1D (Hooper et al., 2017), suggesting the PtdIns(3,5)P_2_ synthesis role of FAB1D in nucleus. Future studies are necessary to determine the precise subcellular localization of FAB1C and FAB1D in plant cells.

## Function of FAB1- and PtdIns(3,5)P_2_-Associated Proteins

Loss of *VAC14* function leads to a lethal male gametophyte phenotype caused by vacuolar reorganization defects during pollen development (Zhang et al., 2018). A similar male gametophyte phenotype is observed in *vac14* and *fab1afab1b* mutants, and bimolecular fluorescence complementation suggests that FAB1A/B and VAC14 physically interact to form a crucial functional protein complex in *Arabidopsis* (Whitley et al., 2009; Hirano et al., 2011; Zhang et al., 2018).

The SAC1 protein has been shown to have PtdIns(3,5)P_2_ phosphatase activity (Zhong et al., 2005), with root hair elongation defects observed in *sac1–2* homozygous T-DNA mutants. In contrast, gain of function SAC1 mutants have longer root hairs than the wild-type, indicating that SAC1 is essential for elongation during root hair morphogenesis (Vijayakumar et al., 2016). Although a direct interaction between FAB1 and SAC1 has not yet been reported, SAC1 and FAB1B have been found to co-localize in the wortmannin-sensitive vesicles of pollen and pollen tubes (Zhang et al., 2018). These data suggest the formation of a complex between SAC1 and FAB1 in *Arabidopsis*.

The data also suggests the presence of PtdIns(3,5)P_2_ phosphatase on the vacuolar membranes of *Arabidopsis*. In fact, yeast FIG 4 orthologs such as SAC2-SAC5 are localized in the vacuolar membrane to catalyze the conversion of PtdIns(3,5)P_2_ to PtdIns3P, thereby controlling the balance between these phosphoinositides and maintaining the morphology of storage and lytic vacuoles (Vermeer et al., 2006; Nováková et al., 2014). *SAC6/SAC1b-, SAC7/SAC1c/RHD4-* and *SAC8/SAC1a*-encoded proteins have been found to be similar to yeast Sac1p and can rescue yeast from Sac1p mutations (Despres et al., 2003), whereas SAC7 and SAC8 are expressed broadly and SAC6 is expressed only in pollen (Despres et al., 2003; Zhong and Ye, 2003). Mutations in the SAC7/RHD4 protein have been associated with aberrant root hairs, while mutant *rhd4–1* roots accumulated higher levels of PtdIns4P *in vivo*, indicating that SAC7/RHD4 has a role in the regulation of PtdIns4P accumulation on the membrane compartments of growing root hair tips (Thole et al., 2008). *sac9* mutants have been shown to accumulate significant levels of PtdIns(4,5)P_2_ and PtdIns4P, and have a constitutively stressed phenotype with shorter roots and extreme cell wall and membrane structure abnormalities (Williams et al., 2005; Vollmer et al., 2011) (Figure 2).

## PtdIns(3,5)P_2_ Effector Molecules

PtdIns(3,5)P_2_ is the least abundant phosphoinositide in eukaryotes (Balla, 2013), and constitutes 0.02% of the total phospholipids in *Arabidopsis* (Hirano et al., 2015). Although low in abundance, PtdIns(3,5)P_2_ has various physiological functions in *Arabidopsis* as a signaling molecule, which cannot be carried out without the help of a range of effector proteins. Recently, a wide variety of effector proteins have been reported to associate with PtdIns(3,5)P_2_. In yeast, it has been demonstrated that autophagy related protein 18 (ATG18) is necessary for autophagy, the cytoplasm-to-vacuole targeting (Cvt) pathway, the delivery of the hydrolase aminopeptidase I to the vacuole, and the early steps of autophagosome formation (Barth et al., 2001; Guan et al., 2001). ATG18 has both PtdIns3P- and PtdIns(3,5)P_2_-binding sites, which are essential for its localization on vacuole membranes and its function as a negative regulator of PtdIns(3,5)P_2_ synthesis (Hasegawa et al., 2017). Eight orthologous ATG18 proteins (ATG18a-ATG18h) have been found in the *Arabidopsis* genome, forming three major subgroups: ATG18a, ATG18c, ATG18d, and ATG18e form a cluster similar with the yeast protein Ygr223c; ATG18b is similar to yeast ATG18, whilst ATG18f, ATG18g, and ATG18h are more divergent forms (Xiong et al., 2005). Among these, ATG18a is required for autophagosome formation during nutrient stress and senescence conditions (Xiong et al., 2005) in coordination with ATG9 (Zhuang et al., 2017).

The plant immunophilin ROF1 containing a FK506 binding domain (FKBP), plays a significant role in the osmotic/salt stress responses of germinating seeds, and interacts directly with PI3P and PtdIns(3,5)P_2_ (Karali et al., 2012). The hydrophilic cation-binding proteins, PCaP1 and PCaP2, are involved in stomatal closure (Nagata et al., 2016) and root hair morphogenesis (Kato et al., 2013). They were also found to preferentially interact with PI(3,5)P_2_ and PtdIns(3,4,5)P_3_, however these interactions were inhibited by association with calmodulin in a Ca^2+^-dependent manner (Nagasaki et al., 2008; Kato et al., 2013). The endosomal protein SNX1 binds to PI3P as well as PI(3,5)P_2_, and is involved in the auxin pathway, regulates endosome maturation (Hirano et al., 2015), PIN2 trafficking (Jaillais et al., 2006), and cortical microtubule organization along with CLIP-associated protein (CLASP) (Ambrose et al., 2013). Vacuolar membrane-localized H^+^-translocating pyrophosphatase (V-PPase) binds to PtdIns(3,5)P_2_, PtdIns(4,5)P_2_, and PtdIns(3,4,5)P_3_ suggesting that PtdIns(3,5)P_2_ may regulate vacuole acidification (Bak et al., 2013). Type-II Rho-related GTPase from plants 10 (ROP10) binds FAB1 and various phosphoinositides [PtdIns3P, PtdIns(3,5)P_2_, PtdIns4P and PtdIns(4,5)P_2_], resulting in localization to and hardening of the root hair shank (Hirano et al., 2018).

## Conclusions and Future Prospects

In plants, the establishment of cell polarity is important for patterning processes. It has been reported that PtdIns(4,5)P_2_ and PtdIns4P 5-kinase, which mediates their interconversion, are specifically enriched in the apical and/or basal polar plasma membrane domains, thereby controlling the polar localization of apical and basal cargoes in specialized cells such as root hairs, pollen tubes, and root cells (Ischebeck et al., 2008; Kusano et al., 2008; Stenzel et al., 2008; Tejos et al., 2014). A recent study showed that FAB1B and PtdIns(3,5)P_2_ are predominantly localized in the plasma membrane of the root hair shank to control cortical microtubule organization and cell wall construction, thereby mediating root hair shank hardening in *Arabidopsis*. These results suggest that PtdIns(3,5)P_2_ and PtdIns(4,5)P_2_ have crucial roles in establishing cell polarity in specialized cells like root hairs. However, the function and significance of the majority of molecules involved in the PtdIns(3,5)P_2_-mediated regulation of cellular processes remains largely unknown. Future studies are required to determine the roles of PtdIns(3,5)P_2_ and its unique regulatory mechanisms in higher plants.

## Data Availability

The datasets for this manuscript are not publicly available. Requests to access the datasets should be directed to mhsato@kpu.ac.jp.

## Author Contributions

TH and MS wrote the review. All authors read and approved the final manuscript.

### Conflict of Interest Statement

The authors declare that the research was conducted in the absence of any commercial or financial relationships that could be construed as a potential conflict of interest.
